# Preclinical Evaluation of Folate Receptor-α Chimeric Antigen Receptor T Cells Exhibits Highly Efficient Antitumor Activity against Osteosarcoma

**DOI:** 10.1158/2767-9764.CRC-25-0086

**Published:** 2025-09-23

**Authors:** Michelle Choe, Danielle Kirkey, Isabel Lira, Grace Hawkins, Melia Blankenfeld, Sarah Menashe, Rhonda E. Ries, Brianna Wrightson, Christina Root, Cyd N. McKay, Jack H. Peplinski, Raisa Glabman, Lara E. Davis, Sanjay V. Malhotra, Richard Gorlick, Elizabeth T. Loggers, Soheil Meshinchi

**Affiliations:** 1 Clinical Research Division, Fred Hutchinson Cancer Center, Seattle, Washington.; 2 Cancer and Blood Disorder Center, Seattle Children’s Hospital, Seattle, Washington.; 3 Translational Science and Therapeutics Division, Fred Hutchinson Cancer Center, Seattle, Washington.; 4 Department of Radiology, Seattle Children’s Hospital, Seattle, Washington.; 5 Preclinical Imaging Shared Resources, Fred Hutchinson Cancer Center, Seattle, Washington.; 6 Comparative Pathology, Fred Hutchinson Cancer Center, Seattle, Washington.; 7 Knight Cancer Institute, Oregon Health & Science University, Portland, Oregon.; 8 Department of Cell, Developmental and Cancer Biology, Oregon Health & Science University, Portland, Oregon.; 9 Division of Pediatrics, The University of Texas MD Anderson Cancer Center, Houston, Texas.

## Abstract

Metastatic and relapsed osteosarcoma remains difficult to treat despite advanced surgical techniques, intensified chemotherapy, and targeted therapies. Adoptive immunotherapies such as chimeric antigen receptor (CAR) T cells are in their nascent stage but remain a viable therapeutic strategy for patients with aggressive solid tumors such as osteosarcoma. Folate receptor-α (FOLR1) has been functionally implicated in osteosarcoma pathophysiology, providing rationale as a potential therapeutic target. We recently advanced a FOLR1-specific CAR T-cell product (FH FOLR1-CART) into a trial in infant acute myeloid leukemia (NCT06609928) and now evaluate this CAR construct against osteosarcoma. We provide comprehensive FOLR1 transcript and protein expression profile in patients with osteosarcoma, cell lines, and patient-derived xenografts, substantiating its significance as a therapeutic target. We further evaluate the *in vitro* and *in vivo* efficacy of FH FOLR1-CART in both standard and patient-derived osteosarcoma cell lines and xenograft models. FOLR1 transcript is expressed in the overwhelming majority of osteosarcoma primary patient specimens, osteosarcoma cell lines, and patient-derived models. FH FOLR1-CART exhibits robust *in vitro* activation and potent cytotoxicity against FOLR1-expressing osteosarcoma cell lines and primary osteosarcoma patient samples. More importantly, FH FOLR1-CART demonstrates potent antitumor activity in both localized and metastatic *in vivo* cell-derived and patient-derived xenograft models, with complete tumor eradication. These results demonstrate a potential therapeutic option for patients with advanced osteosarcoma. FH FOLR1-CART is advancing to an early-phase trial in relapsed/refractory osteosarcoma at Fred Hutchinson Cancer Center and Seattle Children’s Hospital.

**Significance:**

FOLR1 expression has previously been implicated in the pathogenesis of treatment-resistant osteosarcoma. This report demonstrates FOLR1 expression by most osteosarcoma tumors and provides preclinical evidence of robust antitumor activity both *in vitro* and *in vivo* against xenograft osteosarcoma models exhibited by FH FOLR1-CART. These data support ongoing efforts for clinical translation of FH FOLR1-CART to an early-phase clinical trial for patients with aggressive osteosarcoma.

## Introduction

Osteosarcoma remains a difficult disease to treat, with 5-year overall survival (OS) estimated to be less than 30% in the setting of metastatic disease and even lower for relapsed or refractory disease (1–3). Unfortunately, previous trials conducted over the last three decades have failed to demonstrate improved outcomes with intensification of chemotherapy or addition of targeted therapies (4, 5). Adoptive cellular therapies such as chimeric antigen receptor (CAR) T cells for solid tumors are in its nascent stages of development but under active investigation. Given the novelty of these therapies and risk of on-target, off-tumor adverse effects, most current studies are focused on describing the safety profile of cellular products targeting antigens such as GD2, B7-H3, folic acid, EGFR, HER2, and GPC3 (6–9). Whereas the clinical efficacy of these products remains unknown, recent advancements showing sufficient clinical response in adult patients with synovial sarcoma treated with MAGE-A4–specific T-cell receptor T cells have led to the first FDA approval of a cellular therapy for solid tumors (10). This advancement demonstrates the potential success of cellular therapies for solid tumors in the setting of appropriate targeting.

Folate receptor-α (FOLR1) is a folate-binding protein expressed on cellular membranes. It is normally expressed at low levels on the choroid plexus, thyroid, salivary glands, breast, colon, bladder, lung alveoli, and renal proximal tubules (11). Furthermore, FOLR1 expression on normal epithelial cells is restricted to the apical surface, with limited exposure to the bloodstream (11–13). This restriction is lost in the setting of malignant transformation as FOLR1 becomes overexpressed in various solid tumors such as lung, breast, ovarian, and gastric cancers and is associated with more aggressive tumor behavior and poor response to chemoradiation (14–17). As such, FOLR1-targeted therapies are under current investigation for these malignancies, with many studies focused on ovarian cancer (11).

The role of FOLR1 and its implications in therapy-resistant osteosarcoma has previously been described (18). Although the exact role of FOLR1 expressed by osteosarcoma is not well elucidated, there is evidence demonstrating uptake of folate by FOLR1 at a lower concentration gradient than reduced folate carrier, the dominant receptor that binds and transports methotrexate, a mainstay in osteosarcoma therapy (18). Additionally, Yang and colleagues generated patient-derived xenograft (PDX) models from 107 patient samples of osteosarcoma, 84 (78.5%) of which had detectable FOLR1 mRNA expression, some to the level of FOLR1 mRNA expression by an ovarian carcinoma cell line. Thus, this pathway is implicated as an alternative route for folate uptake by tumor cells and allowing tumor growth (18). We recently discovered FOLR1 to be expressed in a highly refractory infant acute myeloid leukemia (AML) driven by the *CBFA2T3::GLIS2* fusion (19, 20). To target this refractory AML, we developed a FOLR1-specific CAR T-cell product (FH FOLR1-CART) using the single-chain variable fragment (scFv) of farletuzumab, CD28 transmembrane, 4-1BB costimulatory domain, and CD3ζ cytotoxicity domain (19). FH FOLR1-CART demonstrated potent antileukemic activity in FOLR1-positive AML models with no hematopoietic toxicity. This CART product is currently being studied in a phase 1 clinical trial (NCT06609928) in relapsed/refractory CBFA2T3-GLIS2 AML (19). In our goal to broaden the use of FH FOLR1-CART to other difficult-to-treat malignancies, we provide potent *in vitro* and *in vivo* evidence of FH FOLR1-CART activity against osteosarcoma.

## Materials and Methods

### Tumor mRNA expression profiling

Whole-genome sequencing data for 2,358 samples from patients with various malignancies was obtained from St. Jude Cloud (21). RNA sequencing expression data from 34,138 samples of patients with various malignancies were obtained and downloaded from the Open Pediatric Cancer Project v15 (pedcbioportal.kidsfirstdrc.org), and 244 samples from the Pediatric Preclinical Testing Consortium [cbioportal.org; now referred to as the Pediatric Preclinical *In Vivo* Testing Consortium (PIVOT); https://preclinicalpivot.org/about-pivot/; refs. 22–24]. Normalized read counts in fragments per kilobase of transcript per million mapped reads (FPKM) were converted to transcripts per million (TPM) using the following formula: TPM = [FPKM/sum (all transcripts in FPKM)] × 10^6^. All conversions were made in R (v4.3.2).

### Primary tumor cells

The U-2 OS osteosarcoma cell line was generously gifted by Dr. Stanley Lee and grown in DMEM (Gibco, cat. # 11965-092) with 10% FBS, 1% L-glutamine (Gibco, 25030-081), and 1% penicillin/streptomycin (Gibco, 15140-122). Cells were routinely tested for *Mycoplasma* contamination using the MycoAlert Mycoplasma Detection Kit [Lonza Corporation (RRID: SCR_000377)]. Cell line identity was validated using the CLA IdentiFiler Plus PCR Amplification Kit [Thermo Fisher Scientific (RRID: SCR_008452)] on an ABI 3730xl Genetic Analyzer [Thermo Fisher Scientific (RRID: SCR_008452)]. The results were analyzed using the Thermo Fisher Connect Microsatellite Analysis software, and allele calls were compared with the entries for the cell line in the ATCC (RRID: SCR_001672) and Cellosaurus (RRID: SCR_013869) databases. Patient-derived cell lines OS766, OS742, OS186, OS457, and OS525 were kindly donated by Dr. E. Alejandro Sweet-Cordero at the University of California, San Francisco. Details about patient characteristics are provided in Supplementary Fig. S1. Patient-derived cell lines were confirmed by short tandem repeat by the Sweet-Cordero Laboratory. PDX tumor cell lines OS9 and OS33 were generously donated by Dr. R. Gorlick at the MD Anderson Cancer Center as solid tumor collected from PDX mice. These cell lines were dissociated to single cell suspension with collagenase II (Gibco, #17101015) prior to flow cytometric analysis. All other patient-derived cell lines were cultured *in vitro* in DMEM with 10% HyClone Bovine Growth Serum [Cytiva (RRID: SCR_023581) #SH30541] and 1% penicillin/streptomycin.

### Lentiviral production and transduction of T cells

Generation of FOLR1-CAR T cells has been previously described (19): CAR T cells were generated by transducing healthy donor T cells (Bloodworks Northwest) with lentivirus carrying the FOLR1 CAR vectors (scFv) derived from farletuzumab to an intermediate-length IgG4 spacer, CD28 transmembrane, 4-1BB costimulatory domain, and CD3ζ cytotoxicity domain. Truncated CD19 separated by 2A short peptide sequence was added and used as a transduction marker. Transduced cells were sorted for CD19 expression using anti-human CD19 microbeads [Miltenyi Biotec (RRID: SCR_008984), 130-050-301]. Sorted cells were further expanded in CTL (with 50 U/mL IL-2) media prior to *in vitro* and *in vivo* cytotoxicity assays. Transduction efficiency was analyzed an FACS analyzer (RRID: SCR_000055; Supplementary Fig. S2).

### Flow cytometry

Expression of FOLR1 on U-2 OS and patient-derived cells was confirmed with anti–FOLR1-PE [BioLegend (RRID: SCR_001134) 908304]. Immunophenotype of T cells was confirmed with anti–CD45-BUV805 [BD Biosciences (RRID: SCR_013311) 612891], anti–CD3-BUV395 [BD Biosciences (RRID: SCR_013311) 564001], anti–CD4-BV605 [BD Biosciences (RRID: SCR_013311) 562658], anti–CD8-BV711 [BD Biosciences (RRID: SCR_013311) 563677], and anti–CD19-APC-eFluor780 [eBioscience (RRID: SCR_003660) 47-0198-42]. Immunophenotype of *in vivo* tissue samples was confirmed with anti–mouse-CD45.1-APC [BD Biosciences (RRID: SCR_013311) 558701] in addition to all antibodies listed previously. BD Symphony II flow cytometer running FACSDiva software [BD Biosciences (RRID: SCR_001456)] was used for analysis. FlowJo software (RRID: SCR_008520) was used for data analysis and visualization.

### *In vitro* cytotoxicity assay by flow cytometry

A measure of 2.5 × 10^4^ U-2 OS cells were co-cultured with CD8^+^ T cells at effector to target (E:T) ratios of 1:1, 2:1, 5:1, and 10:1 in CTL media without IL-2. The co-culture was incubated at 37°C for 6 and 24 hours before harvesting cells. Cells were stained using Fixable Violet Dead Cell Stain Kit [Invitrogen (RRID: SCR_008410) L34955]. BD Canto II flow cytometer (RRID: SCR_018056) running FACSDiva software [BD Biosciences (RRID: SCR_001456)] was used for analysis. FlowJo software (RRID: SCR_008520) was used for data analysis and visualization.

For *in vitro* cytotoxicity assay using patient-derived cell lines, 2.5 × 10^4^ cells (OS186, OS525, OS742, OS766, and OS457) were co-cultured with combined CD4^+^ and CD8^+^ T cells at E:T ratios of 1:1 and 10:1 in CTL media without IL-2. The co-culture was incubated at 37°C for 24 hours before the cells and supernatant were collected. Cells were stained using Fixable Violet Dead Cell Stain Kit [Invitrogen (RRID: SCR_008410) L34955]. BD Symphony II flow cytometer running FACSDiva software [BD Biosciences (RRID: SCR_001456)] was used for analysis. FlowJo software (RRID: SCR_008520) was used for data analysis and visualization.

### Luminex

T cells (1.0 × 10^4^ cells per well, 1:1 CD4 to CD8 T cells) were co-cultured with 1.0 × 10^4^ U-2 OS cells for 24 hours. For experiments using patient-derived cell lines, 2.5 × 10^4^ cells (OS186, OS525, OS742, OS766, and OS457) were co-cultured with combined CD4^+^ and CD8^+^ T cells at E:T ratios of 1:1. The supernatant was collected at 24 hours of co-culture,and analyzed utilizing a Human Luminex Assay to quantify IL-2 [capture antibody by BD Biosciences (RRID: SCR_013311) #555051; detection antibody by BD Biosciences (RRID: SCR_013311) #555040], IFN-γ [capture antibody by Invitrogen (RRID: SCR_008410) #M700A; detection antibody by Invitrogen (RRID: SCR_008410) #M701B], and TNFα [capture antibody by BD Biosciences (RRID: SCR_013311) #551220; detection antibody by BD Biosciences (RRID: SCR_013311) #554511].

### IncuCyte

To further evaluate long-term cytotoxicity, we used IncuCyte Live-Cell Analysis [Sartorius (RRID: SCR_003935)] using e-GFP–expressing U-2 OS cells (1.0 × 10^4^ cells) co-cultured with CD8^+^ T cells at E:T ratios of 1:1, 2:1, and 5:1. Images were acquired every 30 minutes for 24 hours. IncuCyte Cytotox Red Reagent (Essen Bioscience, 4632) was added to culture media at a 1:2,000 dilution for dead cell detection.

### *In vivo* studies

NOD/SCID/γ*c*^*−/−*^ (NSG) mice were obtained from The Jackson Laboratory (RRID: SCR_004633) and housed and bred at the Fred Hutchinson Cancer Center (FHCC). Six- to ten-week-old age-matched females were randomly assigned to experimental groups. Localized femoral tumor was generated with intrafemoral inoculation of 1 × 10^6^ cell line–derived U-2 OS cells transduced with vector encoding eGFP and firefly luciferase separated by a T2A ribosomal skip element to allow for noninvasive bioluminescence imaging (IVIS). Metastatic pulmonary disease was generated with 1 × 10^6^ U-2 OS cells transduced with eGFP and firefly luciferase by tail vein injection. Tumor engraftment was confirmed by IVIS 13 days after tumor cell inoculation. Mice were treated with 5 × 10^6^ FOLR1-CAR or nontransduced (NT) T cells (1:1 CD4^+^ to CD8^+^ T cells) by tail vein injection 14 days after inoculation.

The PDX osteosarcoma model was established and shared by Dr. R. Gorlick. Cryogenically frozen subcutaneous PDX osteosarcoma tumors collected from mice were thawed and cells were dissociated in 6 mg/mL collagenase II (Gibco, 17101-015) for approximately 2 hours. Dissociated tumor cells were passed through a 70-μm strainer, placed into culture media [DMEM (Gibco, 11965-092) + 10% FBS (Corning, 35-010-CV) + 1% penicillin–streptomycin (Gibco, 15140122) + 2 mmol/L L-glutamine (Gibco, 25030081)]. Tumor cells were transduced with vector containing eGFP and firefly luciferase. Cells were maintained in culture for up to 6 weeks. Metastatic pulmonary disease was generated in mice by tail vein injection of 1 × 10^6^ dissociated and transduced PDX-derived cells. Tumor engraftment was confirmed by IVIS (Spectral Instruments Imaging LAGO X) 20 days after tumor cell inoculation. Mice were treated with 5 × 10^6^ FOLR1-CAR or NT T cells (1:1 CD4^+^ to CD8^+^ T cells) by tail vein injection 21 days after inoculation.

Mice were monitored for signs of disease progression and imaged by IVIS or LAGO X every 7 to 14 days. Mice were euthanized when they exhibited symptomatic metastatic disease (tachypnea, hunchback, persistent weight loss, or fatigue). Tissues (blood, femurs, lungs, liver, spleen, and gross tumor) were harvested at necropsy and analyzed for the presence of osteosarcoma.

### Peripheral T-cell collection and detection

Peripheral blood was collected via retro-orbital bleeds into lavender cap K2-EDTA tubes and depleted of red cells using a red blood cell lysis buffer (0.15 mol/L ammonium chloride, 0.01 mol/L sodium bicarbonate, and 0.1 mol/L EDTA). Cells were then immunophenotyped by flow cytometry as described above.

### MRI imaging

Tumor size and progression were evaluated using a preclinical MR scanner (MR Solutions, DRYMAG7.0T) located in the FHCC imaging suite. Each mouse underwent anesthesia using an anesthetic induction chamber with 2% to 2.5% isoflurane distributed from a precision vaporizer. Each mouse was placed on a warm air-heated bed to prevent hypothermia while under anesthesia. Respiration during imaging was monitored via a pneumatic pillow (SA Instruments, ERT gating module). Each imaging session lasted approximately 10 to 30 minutes per mouse. All images were acquired using a mouse body radiofrequency coil. Scanning techniques used are fast-spin echo T2-weighted scans and fast-spin echo T1-weighted scans. Following imaging, each mouse was monitored during recovery from anesthesia in an empty, warm cage.

### CT imaging

Tumor size and progression were evaluated using respiratory-gated high-speed micro-CT lung 3D data sets (Quantum GX2 micro-CT, Perkin Elmer). Following anesthetic induction with 2% isoflurane, each mouse was placed on a bed, and respiration was monitored via gated imaging. Datasets were acquired at 90 kV tube voltage, 88 μA tube current, 36 mm field of view, 72 μm voxel size, and a total scan time of 4 minutes per mouse. A tungsten anode serves as x-ray source. A fixed filter of 0.5 mm aluminum and 0.06 mm copper is placed in front of the exit port to remove low-energy X-rays that contribute to dose but do not improve image quality. One scan gives a radiation dose of 912 mGy. Data were analyzed using the expiratory dataset from the respiratory-gated output. Following imaging, each mouse recovered from anesthesia in a warmed recovery cage.

### Histopathology

Hematoxylin and eosin (H&E) staining was performed by the FHCC Experimental Histopathology shared resource (P30 CA015704). Lung tissue was harvested, fixed in 10% neutral-buffered formalin, embedded in paraffin, and sectioned at 4 to 5 μm onto charged slides. Slides with paraffin sections were baked at 60°C, processed using an automated stainer [Sakura Tissue-Tek Prisma (RRID: SCR_026144)] for H&E staining, and coverslipped with permanent mounting media. Slides were examined by a board-certified veterinary pathologist (R. Gorlick).

### Statistical analysis

An unpaired, 2-tailed Student *t* test was used to determine statistical significance for all *in vitro* studies using U-2 OS and all patient-derived cell lines. Log-rank (Mantel–Cox) test was used to compare Kaplan–Meier survival curves between experimental groups. Statistical significance was defined as *P* less than 0.05.

### Study approval

Experiments were performed after approval by the Institutional Animal Care and Use Committee of the FHCC (protocol 51068) and in accordance with institutional and national guidelines and regulations. The research was performed after approval by the FHCC Institutional Review Board (protocol 9950). The study was conducted in accordance with the Declaration of Helsinki.

## Results

### FOLR1 mRNA expression in osteosarcoma

**Figure 1.**
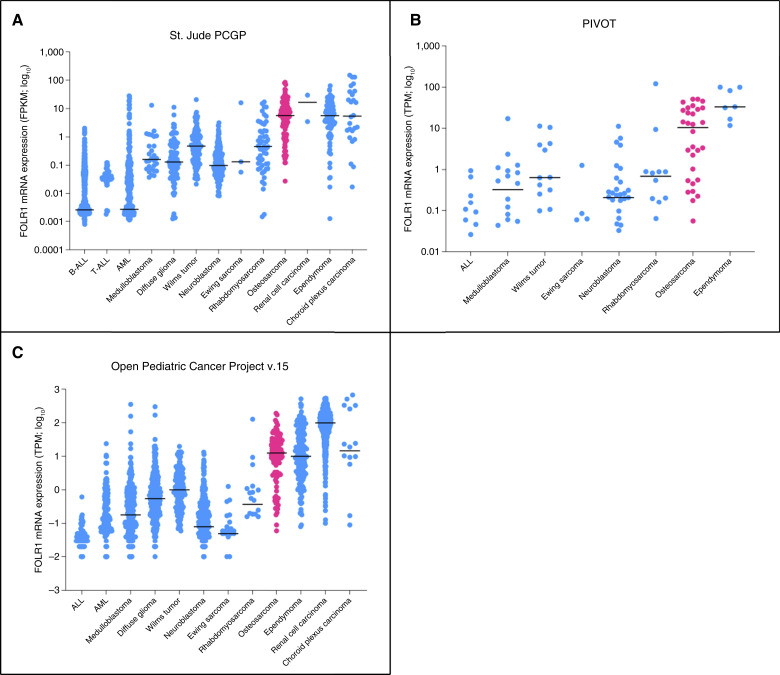
FOLR1 expression by osteosarcoma from various genomic databases. **A,** mRNA expression of FOLR1 in various pediatric malignancies from the St. Jude PCGP database. Increased FOLR1 mRNA expression demonstrated in osteosarcoma, as well as other solid tumors such as ependymoma and renal cell carcinoma. Line across denotes median FPKM. **B,** Data obtained from the PIVOT demonstrate high FOLR1 mRNA expression by osteosarcoma in addition to ependymoma. Line across denotes median TPM. **C,** Data obtained from the Open Pediatric Cancer Project v15 shows increased FOLR1 mRNA expression by osteosarcoma, ependymoma, renal cell carcinoma, and choroid plexus carcinoma. Line across denotes median TPM. ALL, acute lymphoblastic leukemia.

FOLR1 mRNA expression data from primary patient cases were obtained from publicly available St. Jude’s Pediatric Cancer Genome Project (PCGP). The PCGP includes 1,873 patient samples across 16 disease types. Whereas samples from most other tumors demonstrated little or no FOLR1 expression (<2 FPKM), a majority of osteosarcoma tumor samples demonstrated FOLR1 expression with a median FOLR1 mRNA expression of 5.614 (*n* = 113; range, 0–84.12). Other tumor types found to express FOLR1 mRNA include ependymoma, choroid plexus carcinoma, and AML (Fig. 1A; ref. 21). Additional data obtained from the PIVOT, previously known as the Pediatric Preclinical Testing Consortium, and the Open Pediatric Cancer Project v15 reiterate similar findings. There were a total of 244 samples available within the PIVOT database, 30 of which were osteosarcoma samples, expressing a median TPM of 10.448 (range, 0.056–50.976; Fig. 1B). Within the Open Pediatric Cancer Project v15 database, 102 of 31,172 patient samples were obtained from patients with osteosarcoma. These samples had a median TPM of 12.54 (range, 0.0–191.54; Fig. 1C; refs. 22–24). As previously described, FOLR1 expression in normal tissue was tested by IHC on tissue microarray, demonstrating only limited expression in thyroid, renal tubular epithelium, and pulmonary epithelium (Supplementary Fig. S3; ref. 19).

### FH FOLR1-CART demonstrates robust antitumor activity against the FOLR1-positive osteosarcoma cell line U-2 OS *in vitro*

**Figure 2.**
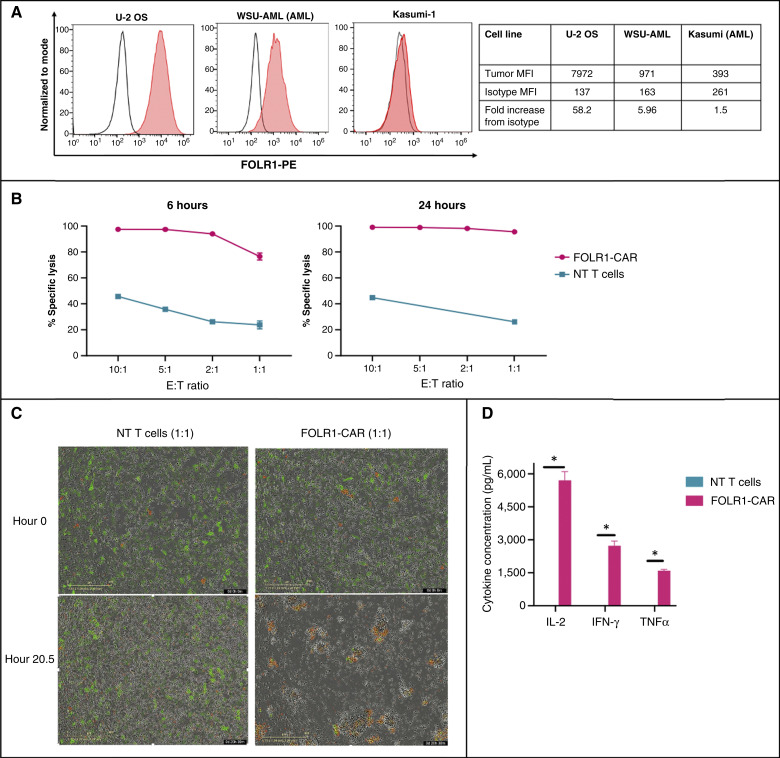
FH FOLR1-CART antitumor activity against U-2 OS osteosarcoma cell line *in vitro*. **A,** FOLR1 expression detected by flow cytometry by U-2 OS, WSU-AML (standard M7, FOLR1-positive AML), and Kasumi (standard FOLR-negative AML). Red, tumor stained with PE-labeled anti-FOLR1; unfilled, isotype control. **B,** Increased FH FOLR1-CAR T cell–induced tumor cell lysis detected by flow cytometry after co-culture at 6 and 24 hours at various E:T ratios, compared with tumor cell lysis induced by NT T cells. **C,** Representative images from the IncuCyte imaging platform at hours 0 and 20.5 of co-culture. Green, live U-2 OS tumor cells; red, dead cells. **D,** Concentration (pg/mL) of secreted IL-2, IFN-γ, and TNFα detected in co-culture supernatant following 24 hours of 1:1 CD8^+^ T cell-to-U-2 OS ratio. *, *P* < 0.0005.

A standard osteosarcoma cell line, U-2 OS was used for initial *in vitro* evaluation. U-2 OS demonstrated high FOLR1 cell surface expression by flow cytometry with a mean fluorescence intensity (MFI) of 7,972 or 58.2-fold increase from isotype control (Fig. 2A). Previously tested AML cell lines used as controls include WSU-AML (positive control) and Kasumi-1 (negative control) which demonstrated 5.96- and 1.5-fold increase in MFI from isotype controls, respectively (19). Having validated FOLR1 surface expression in U-2 OS, we evaluated the antitumor activity of FH FOLR1-CART by co-culturing tumor cells with CD8^+^ FH FOLR1-CART or NT T cells at various E:T ratios ranging from 10:1 to 1:1. FH FOLR1-CART exhibited robust target-specific cytotoxicity ranging from 76.5% (E:T = 1:1) to 97.4% (E:T = 10:1) at 6 hours and 95.5% (E:T = 1:1) to 99% (E:T = 10:1) at 24 hours. In contrast, percent tumor cell lysis upon co-culture with NT CD8^+^ T cells ranged from 23.8% (E:T = 1:1) to 45.7% (E:T = 10:1) at 6 hours and 26.1% (E:T = 1:1) to 44.8% (E:T = 10:1) at 24 hours (Fig. 2B). Live-cell imaging obtained during incubation at hours 0 and 20.5 demonstrated tumor cell death as evidenced by decreased object green area and increased red reagent uptake by dead cells after co-culture with FH FOLR1-CART. Contrastingly, wells with U-2 OS tumor cells co-cultured with NT T cells show continued tumor growth (Fig. 2C). FH FOLR1-CART activation was additionally confirmed by measurement of proinflammatory cytokines IL-2, IFN-γ, and TNFα detected in 1:1 co-culture supernatant collected at 24 hours with no or minimal cytokines detected in the supernatant from NT T cells co-cultured with tumor cells (Fig. 2D). Target specificity against FOLR1 was previously demonstrated by lack of cytotoxicity against FOLR1-negative Kasumi-1 AML cells (Supplementary Fig. S4; ref. 19).

### FH FOLR1-CART demonstrates robust antitumor activity against a FOLR1-positive osteosarcoma cell line–derived xenograft model *in vivo*

**Figure 3.**
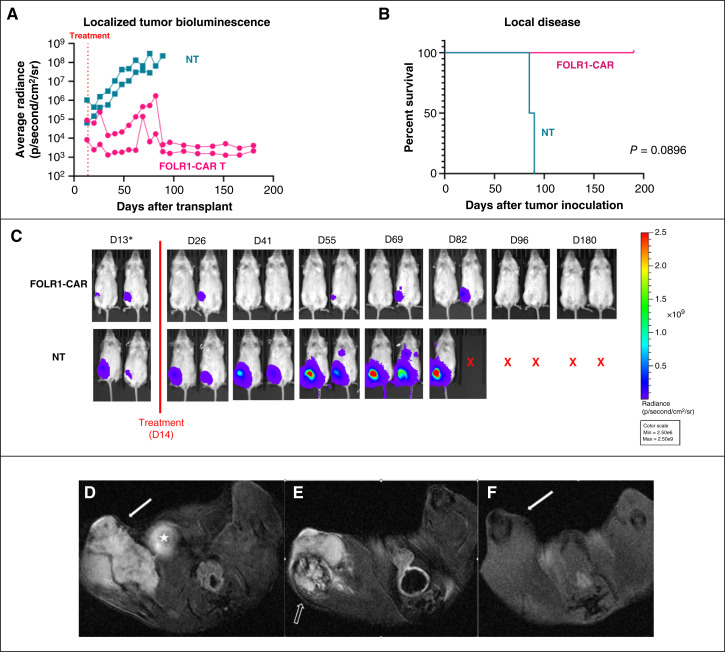
*In vivo* functional evaluation of FH FOLR1-CART in a localized U-2 OS xenograft model. **A,** Average radiance emitted by tumor measured over 180 days from intrafemoral tumor inoculation in mice treated with FH FOLR1-CART vs. NT T cells. **B,** Kaplan–Meier survival curves of xenografts treated with NT or FH FOLR1-CAR T cells. *n* = 2 per group. Statistical differences in survival evaluated using Mantel–Cox log-rank test. **C,** Bioluminescence imaging of localized U-2 OS tumor inoculated intrafemorally in mice treated with NT or FH FOLR1-CAR T cells. Radiance scale indicates progressive osteosarcoma from blue to red. *Day 13 images use a minimum radiance cutoff of 1e6 p/second/cm^2^/sr to show the presence of tumor engraftment prior to treatment. Minimum radiance cutoff of 2.5e6 p/second/cm^2^/sr used for posttreatment images to avoid noise created by excess radiance detected. X indicates death. D, day. **D,** Axial T2-weighted fat-saturated images of mouse femur treated with NT T cells at 84 days after local osteosarcoma cell line inoculation. Note the T2 hyperintense lobulated soft tissue mass (solid white arrow) involving the distal right femur. The bladder (star), noted more medially, also demonstrates T2 hyperintensity. **E,** The bone underlying the mass at 84 days after tumor cell line injection demonstrates abnormal morphology with diffuse marrow signal change and cortical destruction (open arrow). **F,** Axial T2-weighted fat-saturated images of mouse femur treated with FH FOLR1-CAR T cells at 84 days after tumor inoculation. Note the absence of any soft tissue mass or bone marrow signal changes of the distal right femur (solid arrows).

We next investigated FH FOLR1-CART antitumor activity *in vivo* using a localized and metastatic osteosarcoma cell line–derived xenograft (CDX) mouse model. Localized disease was established using U-2 OS by direct intrafemoral inoculation, and tumor engraftment was confirmed by IVIS imaging with comparable average radiance emitted by tumor between two treatment groups (Fig. 3A). Mice that received NT T cells (*n* = 2) demonstrated progressive disease with increased average radiance emission by IVIS, resulting in death by day 90. In contrast, engrafted femoral disease was eradicated and disease control was maintained in mice treated with FH FOLR1-CART (*n* = 2) with 100% OS at day 190 (*P* = 0.0896; Fig. 3B). Mice treated with FH FOLR1-CART demonstrated no signs/symptoms of on-target/off-tumor adverse effects by way of general appearance and weight. Tumor control and gross disease response in treated mice were evidenced by decrease of average radiance emitted by tumor (Fig. 3C). In addition to IVIS, we obtained MRI images of localized disease at multiple time points after T-cell infusion. MRI of the pelvis of the mouse treated with NT T cells demonstrated tumor growth with rapid evolution of soft tissue mass surrounding, and bony destruction of, the involved femur at 84 days after tumor inoculation (Fig. 3D and E). In contrast, MRI images of the mouse treated with FH FOLR1-CART exhibited no tumor growth nor bony destruction at the same time point (Fig. 3F).

**Figure 4.**
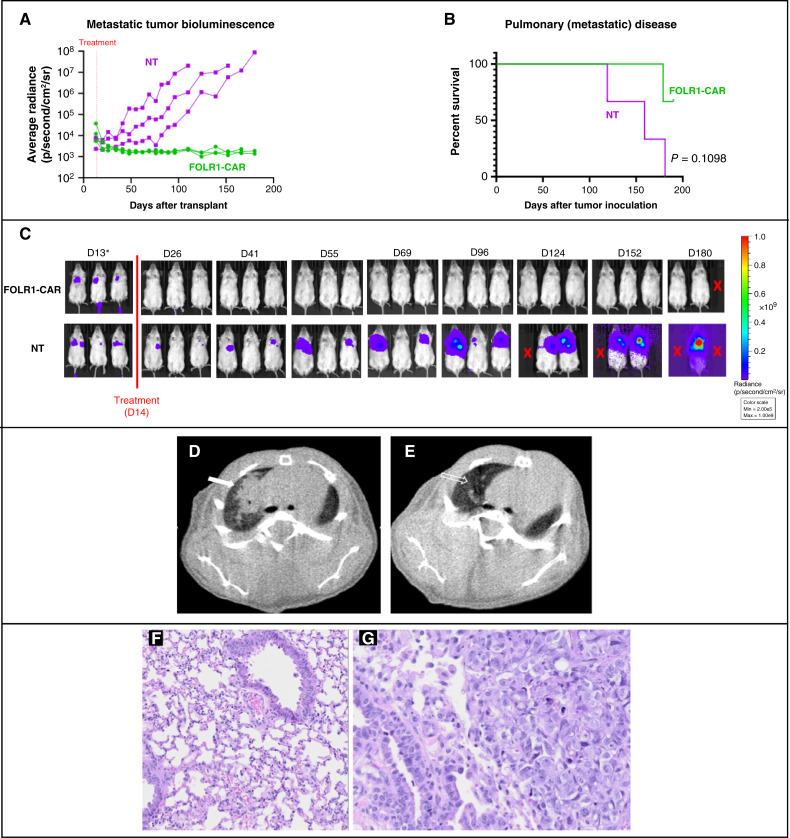
*In vivo* functional evaluation of FH FOLR1-CAR T cells in a metastatic U-2 OS xenograft model. **A,** Average radiance of pulmonary osteosarcoma disease measured over 180 days from tumor inoculation by tail vein in mice treated with FH FOLR1-CART vs. NT T cells. **B,** Kaplan–Meier survival curves of xenografts treated with NT or FH FOLR1-CAR T cells. *n* = 3 per group. Statistical difference in survival evaluated using Mantel–Cox log-rank test. **C,** Biolumiscence imaging of metastatic pulmonary U-2 OS tumor injected by tail vein in mice treated with NT or FH FOLR1-CAR T cells. Radiance scale indicates progressive metastatic osteosarcoma from blue to red. *Day 13 images use a minimum radiance cutoff of 5e4 p/second/cm^2^/sr to show the presence of tumor engraftment prior to treatment. A minimum radiance cutoff of 2e5 p/second/cm^2^/sr was used for posttreatment images to avoid noise created by excess radiance detected. X indicates death. D, day. **D** and **E,** Axial noncontrast CT scan in lung windows in two different mice. **D,** Lobulated soft tissue density (solid arrow) with spiculated margins is present in the right upper lung of mouse treated with NT T cells at 133 days after osteosarcoma injection by tail vein. **E,** Note the normal appearance of the right upper lung, with normal lung markings and the absence of soft tissue mass (open arrow), of mouse treated with FH FOLR1-CAR T cells at same time point. No other lesions were present within the remainder of the lungs. **F,** H&E stain of lung tissue harvested from mouse treated with FH FOLR1-CART at 5× magnification. No tumors or any sign of disease observed within sections taken. **G,** H&E stain of lung tissue harvested from mouse treated with NT T cells at 5× magnification. Expanding and effacing the normal lung are numerous poorly circumscribed, invasive, moderately cellular proliferation of neoplastic mesenchymal cells arranged in trabeculae and streams. Neoplastic cells occasionally surround eosinophilic osteoid and islands of the basophilic cartilaginous matrix.

An *in vivo* metastatic U-2 OS model was established by inoculating tumor cells by tail vein injection, leading to pulmonary metastatic disease. Tumor engraftment was confirmed by IVIS imaging and measured average radiance emitted by tumor (Fig. 4A). Similarly to the localized disease model, mice in the NT control group (*n* = 3) developed progressive disease evidenced by increasing measured radiance that resulted in 100% mortality by 190 days after tumor inoculation. In contrast, mice that received FH FOLR1-CART (*n* = 3) demonstrated improved OS to day 190 (*P* = 0.1098; Fig. 4B). Disease control by day 26 is demonstrated by IVIS imaging with decreased average radiance compared with the control arm at the same time point (Fig. 4C). Additional CT images of mice treated with NT T cells obtained on day 133 after tumor inoculation demonstrated patchy and nodular airspace opacities that grew over time, becoming spiculated and coalescent, largely replacing normal lung parenchyma (Fig. 4D). In contrast, mice treated with FH FOLR1-CART demonstrated normal-appearing lungs, with no airspace opacities or lung masses at any time point after treatment (Fig. 4E), providing further evidence of effective antitumor activity of pulmonary metastatic disease. A single mouse in the treatment arm developed weight loss necessitating euthanasia on day 179, well after the loss of FOLR1-CART persistence. Of note, gross tumor was not found on necropsy. Additionally, lung tissue collected upon euthanasia for all mice were evaluated for osteosarcoma. All three mice treated with FH FOLR1-CART did not demonstrate evidence of disease in all five lung lobes by histology (Fig. 4F), whereas all three mice treated with NT T cells were found to have pulmonary osteosarcoma (Fig. 4G).

Peripheral blood samples from mice were obtained on the day prior to T-cell infusion and days 5, 12, and 55 after infusion. In both localized and metastatic disease models, human T cells were detected in peripheral blood on day 5, with increased population detected in mice treated with FH FOLR1-CART compared with a negligible population in mice that received NT T cells (Supplementary Fig. S5). T-cell contraction was noted by day 12 with undetectable human T cells in both groups by day 55.

### FH FOLR1-CART exhibits antitumor activity against patient-derived osteosarcoma *in vitro* and *in vivo*

**Figure 5.**
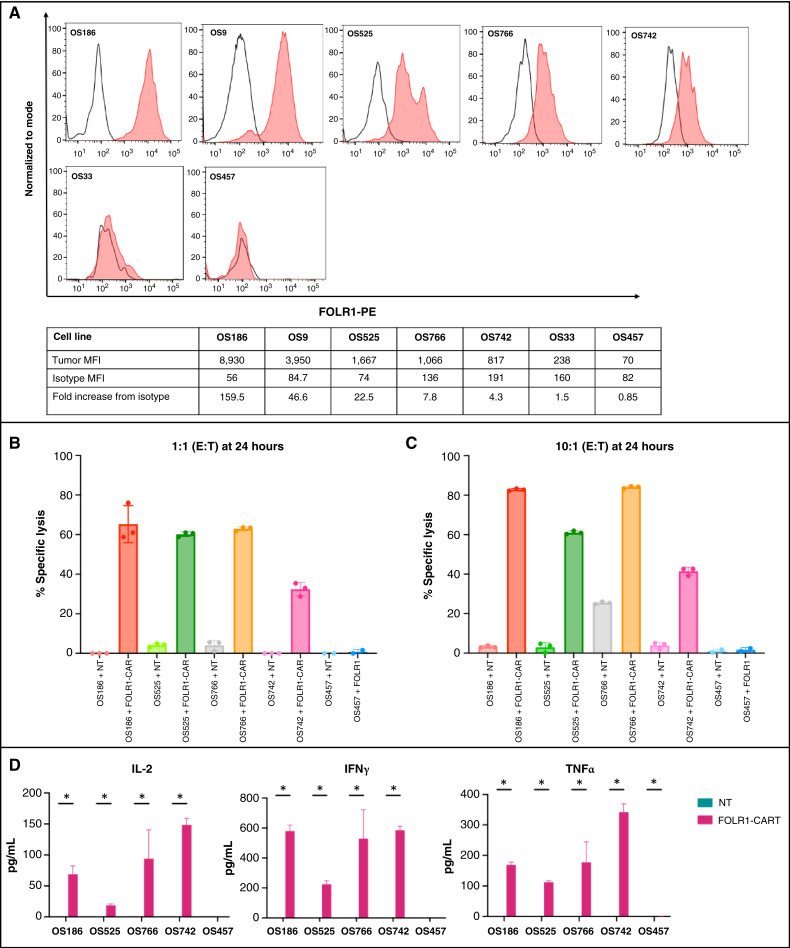
FH FOLR1-CART antitumor activity against patient-derived osteosarcoma cell lines *in vitro*. **A,** FOLR1 expression detected by flow cytometry by a panel of patient-derived osteosarcoma cell lines OS186, OS9, OS525, OS766, OS742, OS33, and OS457. Red, tumor stained with PE-labeled anti-FOLR1; unfilled, isotype control. **B** and **C,** FH FOLR1-CAR T cell–induced tumor cell lysis detected by flow cytometry after co-culture at 24 hours at E:T ratios of 1:1 (**B**) and 10:1 (**C**) in FOLR1-positive patient-derived osteosarcoma cell lines OS186, OS525, OS766, and OS742. No tumor killing was seen in the FOLR1-negative cell line OS457. **D,** Cytokines released by FH FOLR1-CART vs. NT T cells co-cultured with patient-derived osteosarcoma cell lines. Concentration (pg/mL) of secreted IL-2, IFN-γ, and TNFα detected in co-culture supernatant following 24 hours of 1:1 T cell-to-tumor cell ratio. *, *P* < 0.05.

To develop more clinically relevant model systems, we validated the efficacy of FH FOLR1-CART in patient-derived osteosarcoma models. Five of seven osteosarcoma patient-derived cell lines or tissue demonstrated high FOLR1 surface expression by flow cytometry, with MFI ranging from 817 to 8,930 or 4.3- to 159.5-fold increase from isotype control (Fig. 5A). Two of seven patient-derived models, OS33 and OS457, demonstrated minimal FOLR1 expression, with MFI ranging from 70 to 238 or 0.85- to 1.5-fold increase from isotype control. We proceeded to evaluate the *in vitro* antitumor activity of FH FOLR1-CART against four FOLR1-positive (OS186, OS525, OS766, and OS742) and one FOLR1-negative (OS457) PDX cell lines. FH FOLR1-CART demonstrated potent cytotoxicity against all four FOLR1-positive patient-derived cell lines across all E:T ratios and no cytotoxicity in the FOLR1-negative patient-derived cell lines (Fig. 5B and C). FH FOLR1-CART activation upon co-culture with FOLR1-expressing patient-derived cells lines (OS186, OS525, OS766, and OS742) was confirmed by increased secretion of proinflammatory cytokines IL-2, IFN-γ, and TNFα at 24 hours (Fig. 5D). Meanwhile, cytokines were not detected in the supernatant from tumor and NT T cells, as well as FH FOLR1-CART co-cultured with the FOLR1-negative patient-derived cell line, OS457.

**Figure 6.**
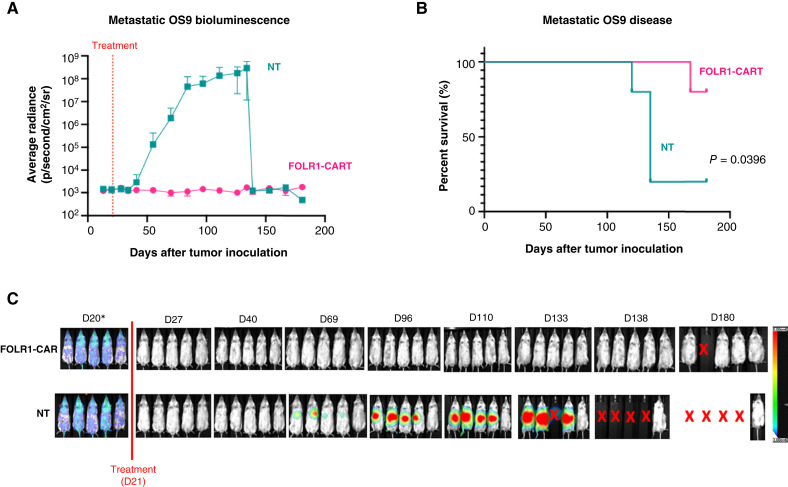
*In vivo* functional evaluation of FH FOLR1-CAR T cells in metastatic PDX model. **A,** Average radiance of pulmonary PDX OS9 disease measured over 180 days from tumor inoculation by tail vein in mice treated with FOLR1-CART vs. NT T cells. **B,** Kaplan–Meier survival curves of xenografts treated with NT or FOLR1-CAR T cells. *n* = 5 per group. Statistical difference in survival evaluated using Mantel–Cox log-rank test. **C,** Bioluminescence imaging of metastatic pulmonary PDX OS9 tumor injected by tail vein in mice treated with NT or FOLR1-CAR T cells. Radiance scale indicates progressive metastatic osteosarcoma from blue to red. *Day 20 images use a minimum radiance cutoff of 3e4 p/second/cm^2^/sr to show the presence of tumor engraftment prior to treatment. Minimum radiance cutoff of 3e6 p/second/cm^2^/sr used for posttreatment images to avoid noise created by excess radiance detected. X indicates death. D, day.

We further validated FH FOLR1-CART in an *in vivo* PDX model. OS9 patient-derived tumor cells were inoculated into NOD/SCID/γ*c*^*−/−*^ mice by tail vein to establish a metastatic PDX model. Tumor engraftment was confirmed by IVIS imaging by day 20 with similar tumor emission noted in the control (NT) group and treated (FH FOLR1-CART) group (Fig. 6A). Mice that received FH FOLR1-CART demonstrated disease control and improved OS with 80% survival at 180 days compared with 20% in the control group treated with NT T cells (*P* = 0.0396; Fig. 6B). Weekly IVIS imaging also showed progressive level of pulmonary disease, evidenced by increasing measured average radiance in four of the five mice, whereas no mice in the FH FOLR1-CART arm demonstrated pulmonary disease (Fig. 6C). One of the CART-treated mice developed weight loss on day 168 and was found to have a FOLR1-positive abdominal tumor upon necropsy, whereas all other FH FOLR1-CART–treated mice remained without evidence of disease. Lung tissue samples were harvested from the remaining surviving treated mice and found to be disease-free on histology. Of note, no significant human T-cell population was detected in mice peripheral blood following T cell administration in both treatment arms on days 6, 12, and 33.

## Discussion

Over the last four decades, multiple randomized trials have failed to identify therapies that provide meaningful improved outcomes in osteosarcoma. This failure is particularly glaring in high-risk osteosarcoma, whether because of failure to respond to primary therapy, relapse after an initial response, or presence of metastatic disease at diagnosis, all of which are associated with unacceptably dismal outcomes (25, 26). Disease control becomes particularly difficult when complete surgical resection is not possible (3). There is a call to develop innovative tumor-targeted therapies that induce durable responses with limited long-term side effects. Development of CAR T-cell therapies for solid tumors has been limited by identification of tumor-specific antigens in order to reduce on-target, off-tumor effects (27). FOLR1 is an attractive target of interest in many solid tumors due to its expression on the surface of tumor cells and low to no expression on normal tissues (11, 14, 28). Folate receptors are a particularly attractive target in osteosarcoma given demonstrated functional biology of the folate pathway and its potential role in therapy resistance (18, 28).

Whereas a previous study describes FOLR1 overexpression in 78.5% of 107 patient-derived osteosarcoma specimens, our analysis of publicly available genomic data through St. Jude’s PCGP, the PIVOT, and the Open Pediatric Cancer Project v15 further demonstrates that most osteosarcoma patient specimens exhibit increased FOLR1 mRNA expression (18, 21–24). Although it is known that mRNA expression does not directly correlate with protein expression (29), we validated FOLR1 tumor surface expression in multiple patient-derived osteosarcoma cell lines and tumors, as detected by flow cytometry. Given these data, we reasoned that osteosarcoma is amenable to targeting with our recently developed FH FOLR1-CART. The FH FOLR1-CART is currently in phase I trial for infant CBFA2T3-GLIS2 AML. Given its remarkable potency against one of the most refractory forms of AML, we conducted preclinical studies to support advancement of FH FOLR1-CART to trial for osteosarcoma.

This article provides detailed *in vitro* evaluation of our FOLR1-CART in a standard osteosarcoma cell line, as well as multiple patient-derived cell lines. *In vitro* tumor cell killing in established patient-derived cell lines demonstrates antigen density–dependent cytotoxicity, with potent tumor cell killing in cell lines with increased FOLR1 expression. Additionally, we completed therapeutic evaluation of FH FOLR1-CART against CDX and PDX models *in vivo*. Although the standard U-2 OS cell line was used as a pilot study to establish both localized and metastatic disease models in mice, the patient-derived tumor cell line OS9 was used to evaluate *in vivo* FH FOLR1-CART activity in a metastatic model. A metastatic PDX model was chosen for further evaluation because of its clinical relevance as patients presenting with unresectable lung metastasis have poorer prognoses, with higher likelihood to pursue alternative therapeutic strategies to surgical resection. Our *in vivo* studies using both U-2 OS and OS9 demonstrate tumor regression and significant OS advantage upon treatment with FH FOLR1-CART. We do note that a single mouse with metastatic U-2 OS treated with FH FOLR1-CART expired prior to our experiment endpoint of 180 days after tumor inoculation. Although the etiology of weight loss is unclear, we did not find any gross or histologic evidence of osteosarcoma. Additionally, the time point at which this mouse developed weight loss is beyond the expected timeline for CAR T-cell toxicity at 179 days after tumor inoculation or 165 days after T-cell administration. Further evidence to support this is lack of T-cell detection in peripheral blood prior to this time point. In our PDX metastatic model, there was one mouse in the control group that did not seem to have adequate tumor engraftment, resulting in prolonged survival. One of the mice in the CART-treated arm developed weight loss by day 168 and upon necropsy was found to have a FOLR1-expressing abdominal tumor, presumed to have developed from engrafted non–GFP-tagged tumor cells, explaining the lack of emitted radiance on IVIS imaging. We confirmed the absence of gross disease in the remaining treated mice at necropsy. Due to concern for potential pulmonary engraftment of non–GFP-tagged tumor cells, lung tissues were harvested and examined after H&E staining. All samples were negative for evidence of disease.

The scFv of FH FOLR1-CART is derived from an anti-FOLR1 humanized mAb, farletuzumab (also known as MORAb-003). Farletuzumab has been previously reported to have specific, high-affinity binding to FOLR1, resulting in decreased cell growth and increased complement-mediated cytotoxicity in testing against ovarian cancer (19, 30). Our *in vivo* experiments demonstrate sustained tumor control in localized as well as metastatic models, without evidence of significant adverse effects. Mice treated with FH FOLR1-CART were able to maintain their weight and general health without signs of tumor growth by physical exam or imaging. However, we acknowledge that there are no published preclinical data demonstrating farletuzumab binding to physiologic FOLR1 in mice. Therefore, these data may not be reflective of FH FOLR1-CART safety in human subjects. Reassuringly, data available from previous clinical studies provide a favorable safety profile for farletuzumab with adverse effects limited to grade 1 or 2 reactions such as headache, nausea, anorexia in adult patients with relapse ovarian cancer (31–34). Other FOLR1-targeting agents such as FDA-approved mirvetuximab soravtansine also did not reveal toxicities specific to off-tumor FOLR1 targeting (11). Therefore, there is currently little reason to be concerned for significant on-target, off-tumor adverse effects.

Our decision to pursue studies with FH FOLR1-CART expressing a receptor based on farletuzumab is further bolstered by a previous study of a first-generation FOLR1-CAR constructed with scFv derived from a murine mAb, MOv18. Whereas patients treated in this study developed cytokine release syndrome, T-cell persistence was noted to be short-lived with limited clinical antitumor response (35). This may be in part due to lack of a costimulatory domain included in CAR T-cell design but also due to immunogenicity against a murine-derived receptor (35). We address the issue of immunogenicity by utilizing a humanized mAb in addition to including a 4-1BB costimulatory domain in the design of FH FOLR1-CART. While recognizing there will be differences in T-cell kinetics in mice versus humans, we demonstrate FH FOLR1-CART expansion in comparison with NT T cells infused into mice, followed by contraction by day 12 in our CDX *in vivo* studies. This pattern is similar to published reports on CAR T-cell kinetics in clinical studies for solid tumors (7, 36). We were unable to detect T-cell expansion in our *in vivo* study using PDXs, which we hypothesize is likely due to lower disease burden at time of treatment, in addition to potential FH FOLR1-CART trafficking to active tumor sites. This is in line with findings reported of CD19-CART expansion in setting of lower disease burden and does not necessarily indicate ineffective CAR T-cell antitumor activity (37). Additional work to improve CAR T-cell persistence by harnessing the immunomodulatory barriers of the solid tumor microenvironment will be required to continue advancement in this field. Further preclinical studies of tumor samples after CAR T cell therapy will provide insight in additional strategies to improve clinical response. Future strategies may include addition of engineered cytokine release by activated CAR T cells to further support cytolytic activity, as well as other strategies to improve tumor susceptibility to cellular therapies (9, 38).

FH FOLR1-CART is currently under investigation in a phase 1 dose-finding clinical study for pediatric patients with FOLR-positive relapsed or refractory AML (NCT06609928). While we await further clinical data on feasibility, dosing, and safety, we look to expand the indication for use in other difficult-to-treat malignancies. Our preclinical data support FH FOLR1-CART antitumor activity against CDX as well as PDX osteosarcoma models. As such, we plan to expand further clinical evaluation of FH FOLR1-CART in an early-phase clinical trial for patients with FOLR1-expressing, chemorefractory, or unresectable metastatic osteosarcoma.

## Supplementary Material

Supplementary Figure S1 Supplementary Figure S1. Clinical characteristics of PDX specimens generously provided by the lab of Dr. E. Alejandro Sweet-Cordero at the University of California, San Francisco.

Supplementary Figure S2 Supplementary Figure S2. FOLR1 CAR construct and lentiviral transduction efficiency.

Supplementary Figure S3 Supplementary Figure S3. Expression of FOLR1 in normal tissues.

Supplementary Figure S4 Supplementary Figure S4. FOLR1-negative control, Kasumi-1.

Supplementary Figure S5 Supplementary Figure S5. In vivo human T cell detection by flow cytometry.

## Data Availability

Whole-genome sequencing data for pediatric relapse tumor samples used for analysis in this study were obtained from St. Jude Cloud (https://www.stjude.cloud; accession # SJC-DX-1001; ref. 21). FOLR1 mRNA expression data are publicly available as described above. The data generated in this study are available in the article, supplemental files, or upon request from the corresponding author.
