# Supplementary figures and images for: Preclinical Evaluation of Folate Receptor-α Chimeric Antigen Receptor T Cells Exhibits Highly Efficient Antitumor Activity against Osteosarcoma
Source: Cancer Res Commun. 2025 Sep 23;5(9):1701–13. doi: 10.1158/2767-9764.CRC-25-0086 (PMC12455178; doi:10.1158/2767-9764.CRC-25-0086)

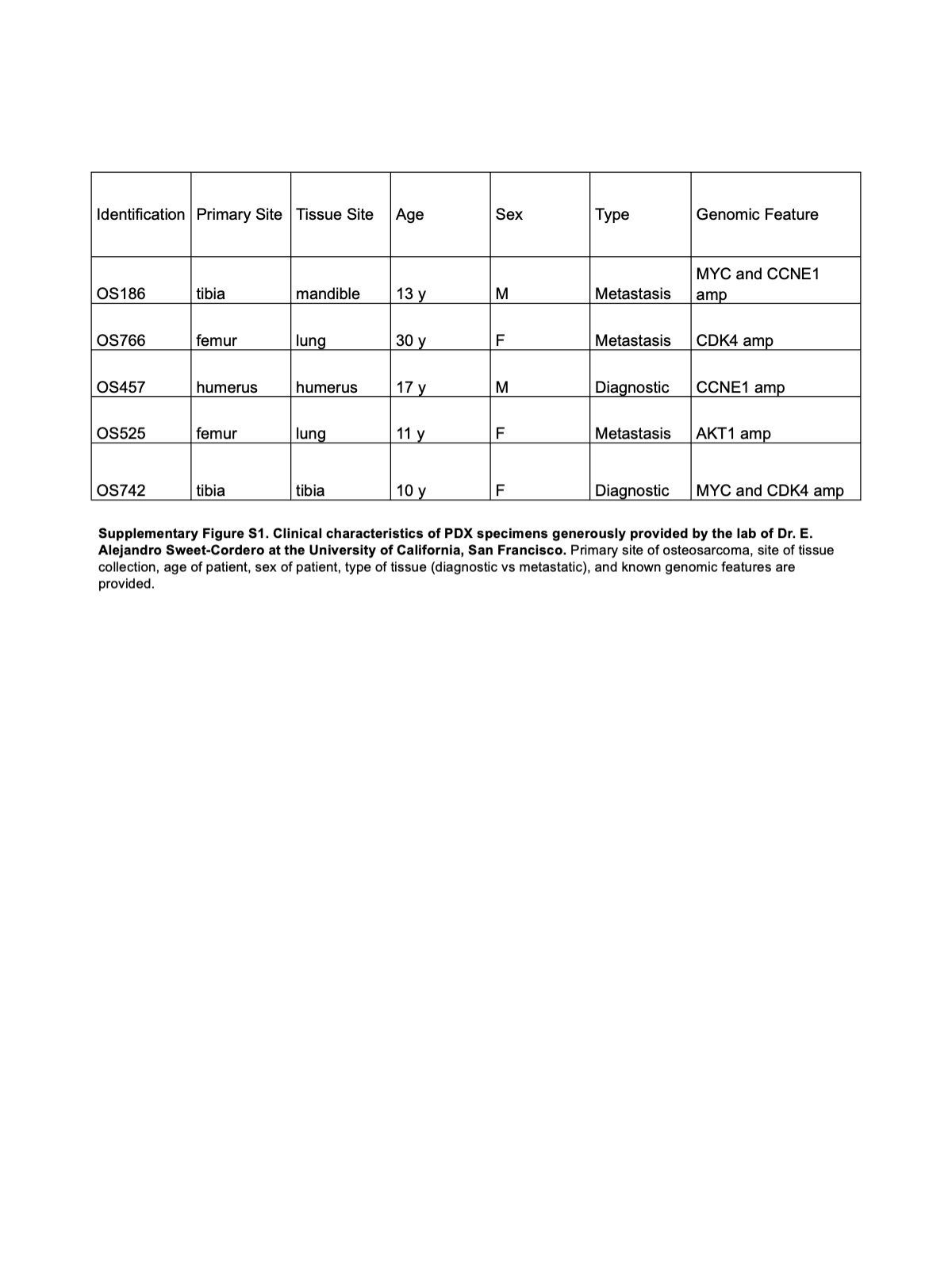

Supplement: Supplementary Figure S1 — Clinical characteristics of PDX specimens generously provided by the lab of Dr. E. Alejandro Sweet-Cordero at the University of California, San Francisco. [file crc-25-0086_supplementary_figure_s1_suppsf1.png]

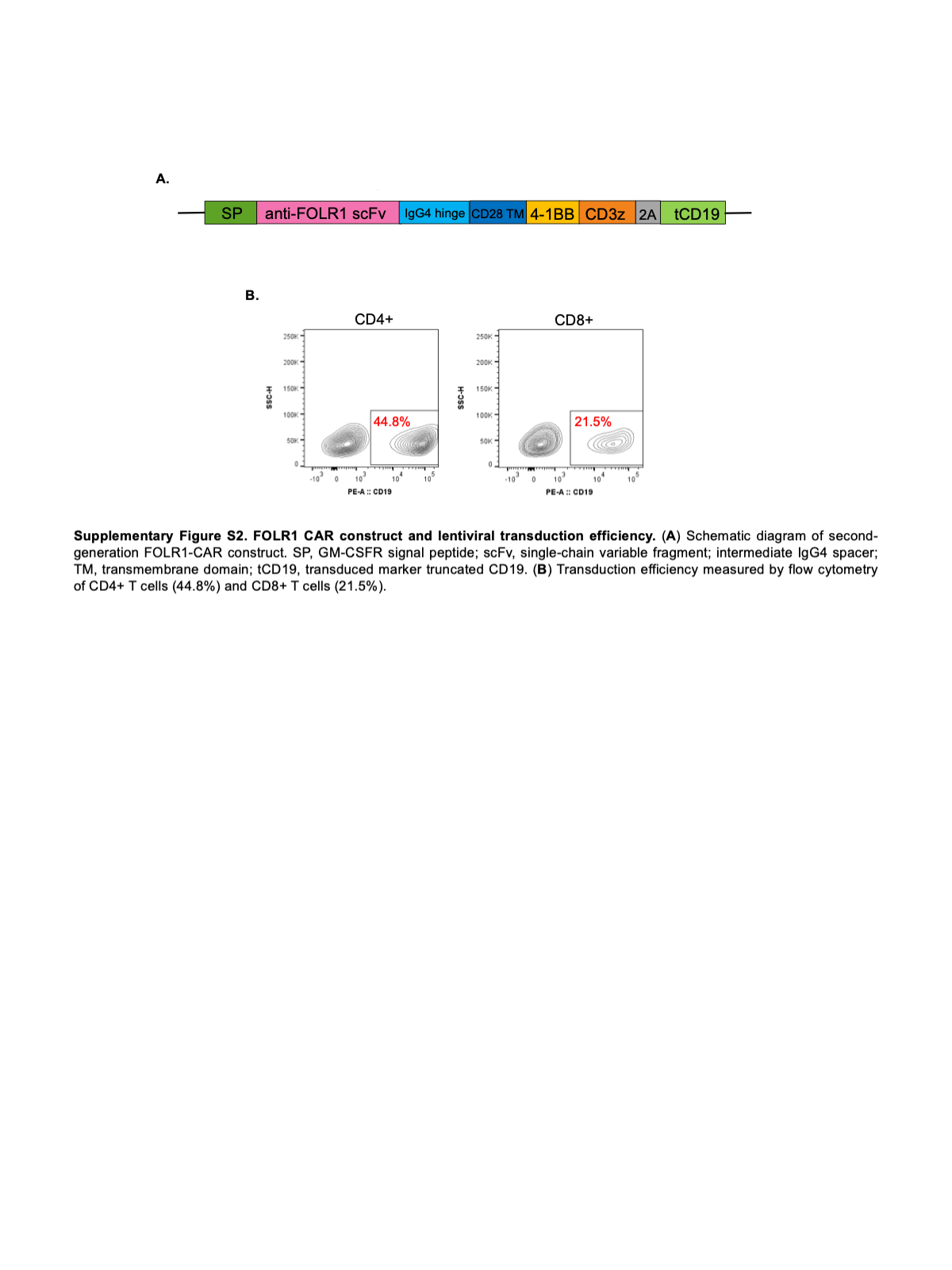

Supplement: Supplementary Figure S2 — FOLR1 CAR construct and lentiviral transduction efficiency. [file crc-25-0086_supplementary_figure_s2_suppsf2.png]

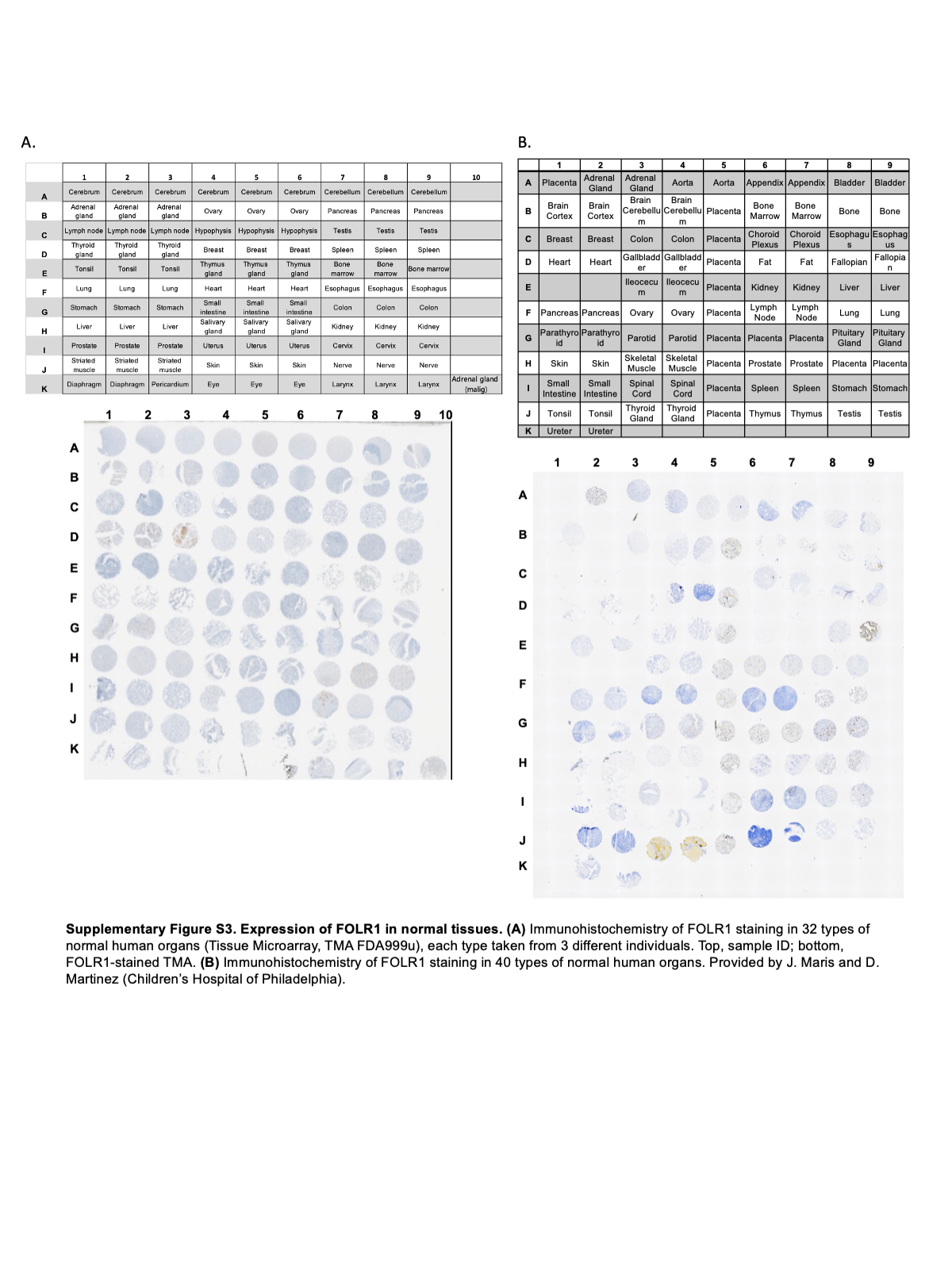

Supplement: Supplementary Figure S3 — Expression of FOLR1 in normal tissues. [file crc-25-0086_supplementary_figure_s3_suppsf3.png]

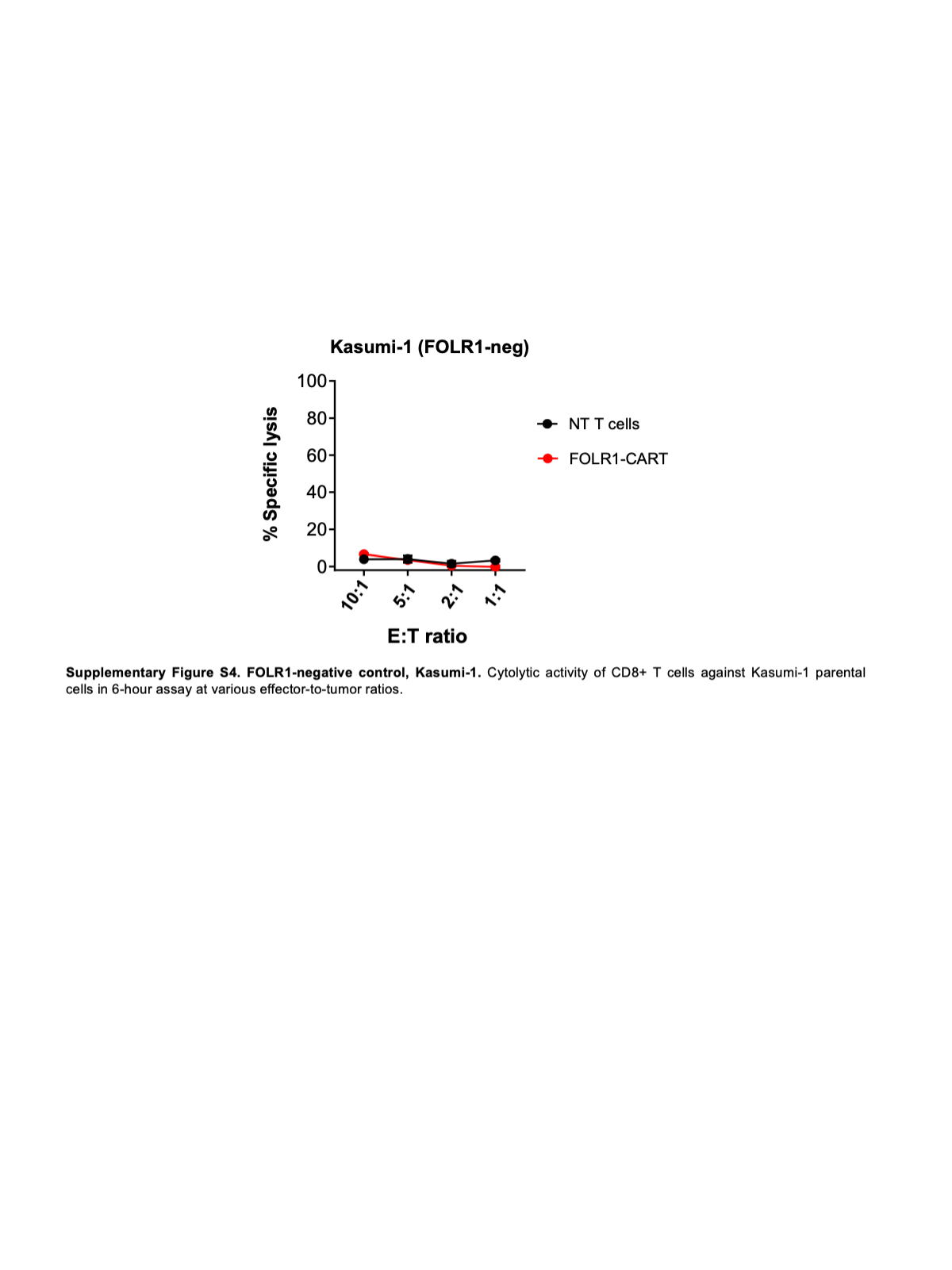

Supplement: Supplementary Figure S4 — FOLR1-negative control, Kasumi-1. [file crc-25-0086_supplementary_figure_s4_suppsf4.png]

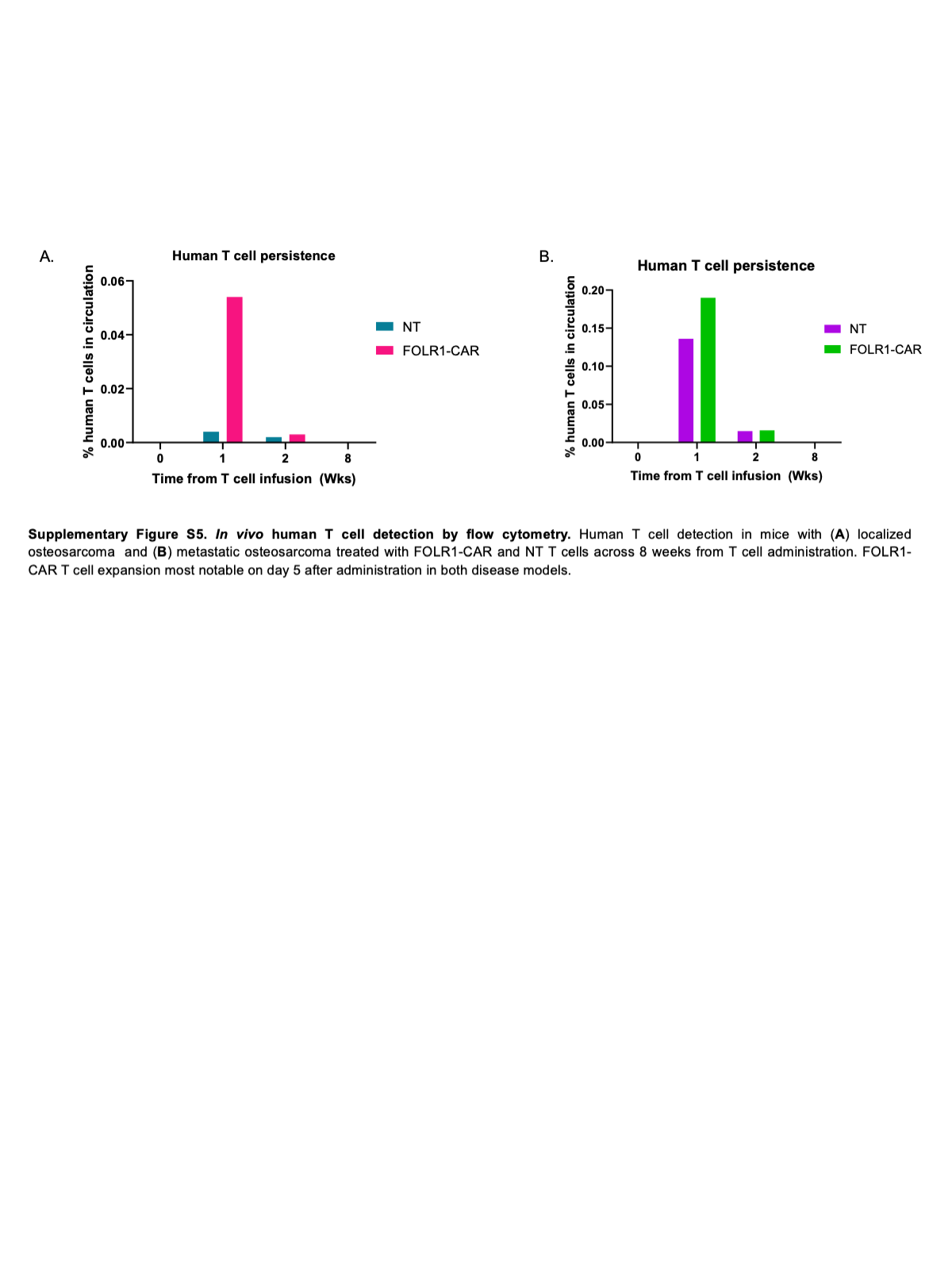

Supplement: Supplementary Figure S5 — In vivo human T cell detection by flow cytometry. [file crc-25-0086_supplementary_figure_s5_suppsf5.png]
